# A Novel Dictionary Based Computer Vision Method for the Detection of Cell Nuclei

**DOI:** 10.1371/journal.pone.0054068

**Published:** 2013-01-24

**Authors:** Jonas De Vylder, Jan Aelterman, Trees Lepez, Mado Vandewoestyne, Koen Douterloigne, Dieter Deforce, Wilfried Philips

**Affiliations:** 1 Department of Telecommunications and Information Processing, iMinds, Ghent University, Ghent, Belgium; 2 Laboratory for Pharmaceutical Biotechnology, Ghent University, Ghent, Belgium; University of Navarra, Spain

## Abstract

Cell nuclei detection in fluorescent microscopic images is an important and time consuming task in a wide range of biological applications. Blur, clutter, bleed through and partial occlusion of nuclei make individual nuclei detection a challenging task for automated image analysis. This paper proposes a novel and robust detection method based on the active contour framework. Improvement over conventional approaches is achieved by exploiting prior knowledge of the nucleus shape in order to better detect individual nuclei. This prior knowledge is defined using a dictionary based approach which can be formulated as the optimization of a convex energy function. The proposed method shows accurate detection results for dense clusters of nuclei, for example, an F-measure (a measure for detection accuracy) of 0.96 for the detection of cell nuclei in peripheral blood mononuclear cells, compared to an F-measure of 0.90 achieved by state-of-the-art nuclei detection methods.

## Introduction

Cell nuclei are extensively studied objects in microscopic biology. This is because they are easily visualized independent of the type of cells, typically using a fluorescent staining, and contain relevant biological information for a wide range of applications, e.g. cell division in tumors, root growth in plants, embryonic development, fetal microchimerism in autoimmune (thyroid) diseases etc. [1]–[5]. In the case of fetal microchimerism in autoimmune diseases, automatic detection of fetal peripheral blood mononuclear cells (PBMCs) in the maternal circulation can shed light on the potential role of fetal cells in these diseases [6]. It is very labour-intensive to locate one or more fetal cells in a population of millions of maternal cells. Staining of the male fetal cells using Fluorescence in Situ Hybridization (FISH) with subsequent 4′,6-diamidino-2-phenylindole dihydrochloride (DAPI) staining and automatic detection of all cell nuclei on a slide, can facilitate the detection of fetal cells.

Due to the biological importance of cell nuclei, several automated detection methods have been proposed in the past. These methods can generally be categorized into two groups: edge based and intensity based. The first group starts by detecting edges and fits a specific shape model to them [7]–[10]. Both the use of binary edge detectors and edge strength maps have been proposed. The performance of these methods strongly depends on the quality of the edge maps, which is not always sufficient to detect individual nuclei in case of clustered nuclei. The second group first segments any fluorescent staining from the background. In contrast to isolated nuclei which can be detected using connected component labeling, detection of an individual nucleus in a cluster of touching nuclei requires an extra step. This is mainly done by requiring the detected segments of the cell nuclei to have a convex shape [3], [11]–[13]. To overcome this problem, joint segmentation of cell nuclei and cell wall or cytoplasm was proposed, assuming each cell can only contain a single nucleus [14], [15]. However, this approach limits the application area since multiple staining is necessary. Moreover, the methodology was only validated for specific cell networks. A different approach is to assign a confidence measure for each detected nucleus, thus only analyzing “reliable” nuclei detections. This however has the risk of rejecting specific subpopulations of cells or specific phenotypes [5].

Both approaches, edged based and intensity based, are non optimal since the detection and recognition steps are treated independently: the second step can only use the result of the first step, instead of all available information, i.e. the complete image. Thus, incorrectly segmented pixels or edges can have a big influence on the final detection of individual nuclei. Because both tasks can be mutually beneficial [16], [17], we propose a novel method which jointly optimizes the foreground, i.e. pixels of interest, classification and the separation of segments into individual nuclei. The decision of segmenting a pixel as foreground is based both on intensity and on the likelihood that the pixel is part of a nucleus. Such a joint optimization has been proposed in [16], [17], where individual circular objects are modeled using Markov random fields. The approach shows good results for circular objects of the same size, but analyzing objects with different sizes is non-trivial. Furthermore, their joint model is optimized using meta-heuristics such as simulated annealing, so it is dependent of the initialization of the optimization process. In this work we propose a different model which also jointly optimizes the detection of relevant pixels and the detection of individual nuclei. The proposed method fits within the general active contour framework, but with a novel shape prior specifically developed for the detection of cell nuclei. We propose a shape regularization term that exploits the regular shape of cell nuclei, penalizing segments which strongly deviate from the expected shape. In this work, we model a nucleus as a disk. This allows us to build a dictionary consisting of binary images that correspond to the segmentation result of a single nucleus with a predefined radius and location, called atoms. Any segmentation result of an image containing multiple nuclei, can be approximated linearly as a superposition of atoms. We will use this approximation as a new regularization term in the active contour framework. The segmentation result is calculated by minimizing a convex energy function, such that the active contour is invariant to initialization [18], [19]. The proposed joint convex optimization approach results in more accurate nuclei detection, especially for realistic segmentation problems, where cell nuclei can have different sizes.

## Materials and Methods

### 0.1 Ethics Statement

This study was approved by the Ethics Committee of Ghent University (B67020095877), Belgium, and written informed consent was obtained from all participants.

### 0.2 Cell Culture and Transfection

The proposed method was tested on the detection of peripheral blood mononuclear cells (PBMCs). PBMCs were isolated from a healthy volunteer's EDTA blood sample by density gradient centrifugation on Ficoll-Paque Plus (GE Healthcare, Diegem, Belgium) according to the manufacturer's instructions. 300.000 PBMCs were cytospun on a Poly-L-lysine coated slide (DAKO) as previously described [20]. The slides were air dried and fixated for 1 minute in 70% EtOH. After air-drying, the slides were mounted with antifade Vectashield mounting solution (Vector Labs, Burlingame, CA, USA) containing 4′,6-diamidino-2-phenylindole dihydrochloride (DAPI, 400 ng/ml, Sigma-Aldrich) to counterstain all nuclei on the slide. A coverslip was applied.

### 0.3 Image Acquisition

Image acquisition was carried out with the AxioVision multichannel fluorescence module and the AxioCam MRm camera (Carl Zeiss). Cell nuclei were visualized using Zeiss filter set no. 49 (G 365 nm, FT 495, BP 445/50). Slides were scanned at 20× magnification using a Carl Zeiss short distance Plan-Apochromat ® objective [4]. Images were acquired and were stored as tiff-files.

### 0.4 Image Analysis

#### 0.4.1 Notations and Definitions

In this paper we will use specific notations and definitions. We briefly summarize the notations and symbols used in this work.

Let 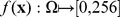 represent the image intensities, with 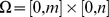, and 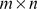 the dimension of the image. Let S be the set of all functions of the form 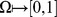, We will represent the segmentation result as a function 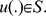 A pixel 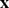 that corresponds to a nucleus, will be represented by one, whereas background pixels are represented by zero, i.e.
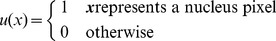
(1)


The details on how to calculate this segmentation result, 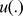, will be thoroughly described in the segmentation subsection. Furthermore we will use the following gradient operator, inner product and norm notations:
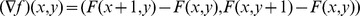


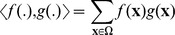


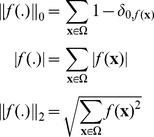
where 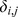 represents the Kronecker delta, which is equal to one if 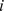 and 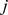 are equal and is equal to zero in all other cases.

#### 0.4.2 Preprocessing

Automatic cell nuclei detection is hampered by a number of factors such as non-uniform lighting, blur, clutter, etc. In order to improve the results of our method, we propose two preprocessing steps to minimize the influence of these degradations. First, the image is normalized in order to remove any differences in intensity:
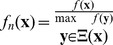
(2)where 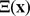 represents all pixels within a distance 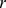 of the pixel 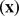. The chosen distance depends on the image resolution and on the nuclei density. 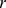 should be chosen in such a way that this disk contains at least one nucleus, since this nucleus' intensity is used as an estimator of the local light intensity.

Furthermore, a gamma correction is applied, i.e.
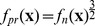
(3)


**Figure 1:**
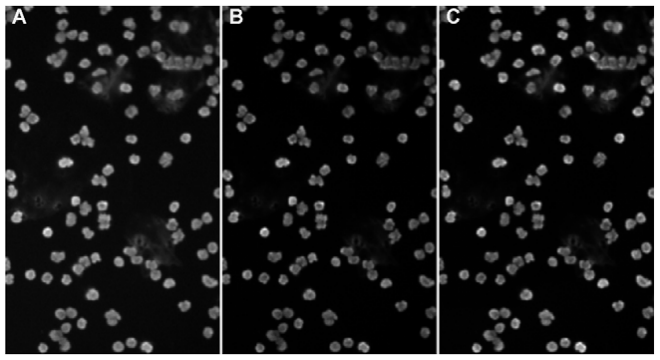
An example of the preprocessing steps. (A) the raw image (B) the image with gamma correction applied, (C) The full prerprocessing applied, i.e. normalization+gamma correction.

This gamma correction suppresses low intensity dyeing due to cell apoptosis. Figure 1 shows an example of the preprocessing step. As can be seen in Fig. 1.b, gamma correction alone results in lower intensity in specific regions, e.g. the nuclei in the top of the micrograph are darker than those near the center. The combination of normalization and gamma correction results in good contrast, bright nuclei, while suppressing dye coming from dead cells (Fig. 1.c).

#### 0.4.3 Segmentation

In [18] a segmentation method was proposed where the segmentation result is calculated by minimizing the following energy function:
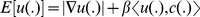
(4)With
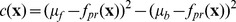
(5)where 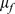, 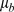 are respectively the expected foreground and background intensity and 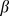 is a weighting parameter used to tune the influence of the data-fit term in relation to the total variation regularization. Note that if 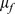 and 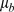 are constant, e.g. calculated from a training data set, this energy is convex. This allows to calculate a global optimizer using efficient optimization techniques such as Split Bregman or primal dual optimization [21]. Chan et al. proved that the solution 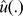 is well-defined as the solution of a convex energy function if 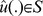, i.e. if the co-domain of 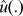 is equal to the convex region 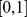. This results in the following optimization problem:
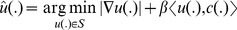
(6)Furthermore, this formulation relates to the popular and widely used active contour without edges (ACWE) [18], [22]. The steady state of the gradient flow corresponding to the energy function in eq. (4) coincides with the steady state of the gradient flow of the original ACWE model, i.e. an optimum of this convex energy is also an optimum of the original ACWE energy function. Note that 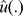 is not necessarily unique, i.e. there can exist different 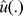, that minimize the energy function in eq. (4). The function 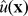 can have any value between 0 and 1, thus the found active contour does not have to represent a crisp segmentation. A binary segmentation result can be obtained by thresholding 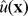, i.e.
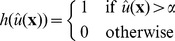
(7)for some 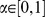. In [19], [23] it is shown that 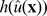 itself is also a global minimizer for the energy in eq. (4) and by extension for the energy function of the ACWE model.

The energy function in eq. (4) aims to remove noisy segmentation pixels by regularizing the energy function using total variation. This regularization is useful if pixels are incorrectly classified, i.e. background pixels detected as foreground or vice versa, due to noise in the image. In microscopic images however, incorrectly detected nuclei are often caused by clutter in the image, e.g. dead cells or bleed-through from other fluorescent channels. This is not solved using total variation since these incorrectly detected nuclei are natural objects, i.e. they have smooth boundaries. Therefore, a number of shape based regularization terms have been proposed [24]–[28]. However these shape priors are limited to images with only a single object of interest with a specific shape. We propose a regularization term that exploits the regular shape of cell nuclei, penalizing segments which strongly deviate from the expected shape, while not constraining the number of nuclei.

In this work, a nucleus is modeled as a disk. For a given radius, r, and location, 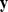, we can calculate the ideal 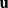, i.e. a binary image where the pixels within a distance r of 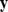 are equal to one and all other pixels are equal to zero:
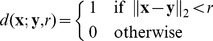
This is of course under the assumption that there is only one nucleus in the image. We will refer to each of these possible segmentation results as *atoms*. In most applications however the image does contain multiple nuclei. Even the number of nuclei is typically not known. Therefore we model the unknown segmentation 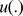 as a superposition of disks, i.e.
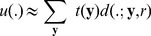
(8)where 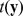 represents a coefficient which expresses a nucleus centered at location 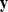. Note that this representation expects a predefined diameter of a cell nucleus. However the size of a nucleus is generally not fixed, but can be considered to an interval 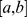. Therefore we can extend eq. (8) to approximate 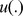 using a dictionary of atoms corresponding to discrete range of nuclei sizes:
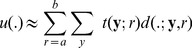
(9)with 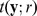 corresponding to the presence of a nucleus of size r at location 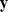. Note that this linear combination penalizes overlapping nuclei, which is desirable since a pixel can only correspond to a single nucleus. A good segmentation is one which consists of a small number of atoms. This sparsity constraint can be used as a new regularization term:
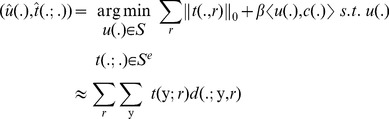
(10)with e the number of disc sizes considered, i.e. 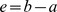. The energy term minimized by 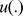 is based on a 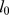 norm which comes down to calculating the number of nuclei, i.e. the number of non-zero elements in 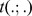. The 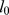 norm is non-convex, hampering optimization. Fortunately the 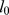 norm can be approximated by the 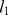 norm which is the closest convex norm to 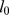. In [29] it is shown that this approximation of an 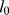 norm gives good results for the application of compressed sensing. This new prior results in the following active contour:
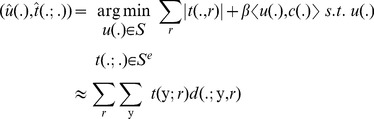
(11)


In order to optimize the constrained problem in eq. (11) the problem is approximated by adding the constraint in the form of a quadratic term, resulting in the following unconstrained optimization problem:
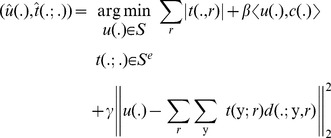
(12)Where 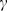 is a weighting parameter. Note that this only approximates the constraint in eq. (9). Although there exists efficient techniques to enforce this constraint exactly, e.g. augmented Lagrangian or Bregman methods, we propose to use the approximation in eq. (12) instead. This allows the active contour to detect nuclei whose shape slightly deviates from the circular model or to detect partially overlapping nuclei. Given the convexity of eq. (12), this problem can be solved by iteratively optimizing for 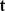 and 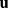 independently, i.e.
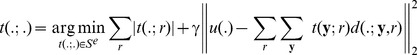
(13)

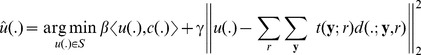
(14)The problem in eq. (13) can be solved using Newton's method, which iteratively updates 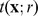 using the following scheme: 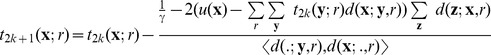
(15)where the subscript index represents the iteration step. The constraint that 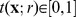 can be approximated by adding a barrier function to eq. (13). For this purpose we propose the use of a piecewise linear barrier function [21]:
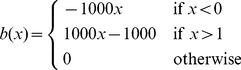
(16)


This barrier function has the benefit of penalizing values out of the interval 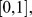 while not giving preference to any specific value inside the interval. The piecewise linear barrier function is prone to overshooting using the Newton-Raphson method, i.e. if 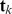 is greater than 1, then 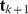 would be equal to 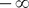, whereas if 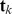 is less than 0, it would result in 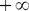. However given the specific nature of this barrier function, with the minima corresponding to the roots of the function, it is possible to minimize this by searching for the roots using the Newton-Raphson optimization scheme. This results in the following updating step:
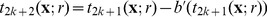
(17)with
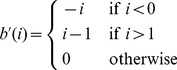
(18)


The optimal 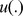 in eq. (14) can be found by solving a set of Euler-Lagrange equations. For an optimal 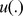, the following optimality condition should be satisfied:
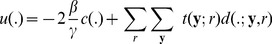
(19)The solution of eq. (19) is unconstrained, i.e. 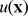 does not have to lie in the interval 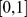. However minimizing eq. (14) in 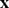 is equivalent to minimizing a quadratic function. So if 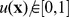 then the constrained optimum is either 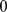 or 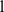, since a quadratic function is monotonic in an interval which does not contain its extremum. So the constrained optimum is given by:
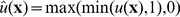
(20)


#### 0.4.4 Detection

**Figure 2:**
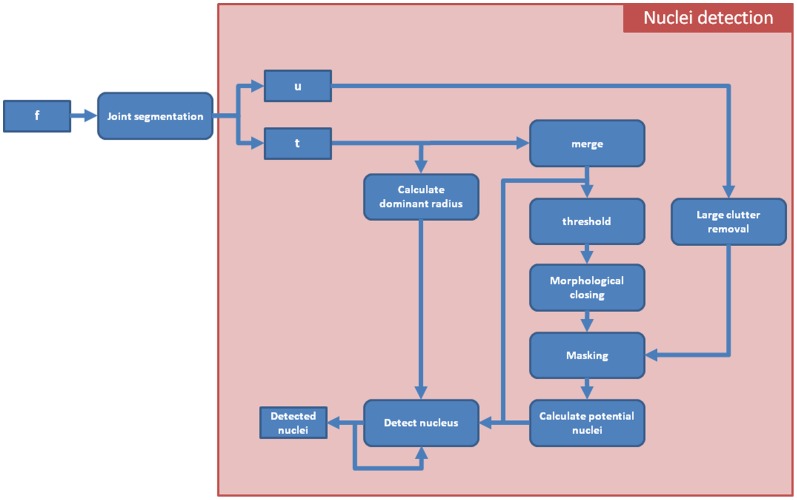
The work flow of the detection algorithm.

**Figure 3:**
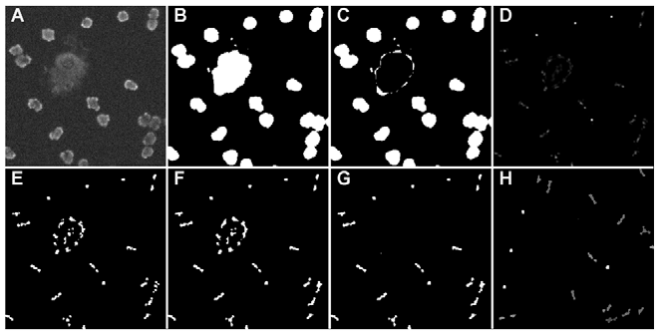
The different steps in the detection algorithm. (A) the original image, (B) the segmentation result, (C) large clutter removal, (D) the merged values for t, corresponding to the centroids of the disks used for the reconstruction of the segmentation result, (E) a binarization of (D), (F) show the morphological processing of (E), (G) is the masked version of (F) with (C) used as mask, (H) represents the intensity of each connected component by it's score, i.e. a measure of likeliness that that component corresponds to the centroid of a nucleus.

By adding a sparsity constraint on 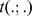, we penalize representations which use more atoms than necessary. The function 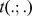 can be used to detect the centroids of the cell nuclei. However by using the 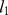 norm as an approximation of the 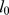 norm it is not possible to get the nuclei centroids explicitly from 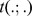, a special detection algorithm is required. A schematic overview of the detection method is shown in Fig. 2. Since disks of multiple sizes can add to the representation of a nucleus in the segmentation result, we first detect the dominant radius for each pixel. Each pixel has a number of corresponding atoms, each representing a nucleus of different size. The radius corresponding with the atom with the highest value for a specific pixel is considered the dominant radius of that pixel, i.e. 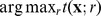. Since the most important information is the location of a cell nucleus, a merging step is applied such that all 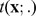 weights corresponding to the same pixel location are combined by summing them together. This is done in parallel with the calculation of the dominant radius. The merging step results in a new image, 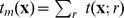, where high intensities occur at the centers of nuclei (shown in Fig. 3(d)). In order to get crisp detections, the new image is converted to a binary image by thresholding it (Fig. 3(e)). Due to small deviations in the shape of cell nuclei, it is possible that a single nucleus corresponds to multiple connected components in this binary image. Since these components are located in each other's vicinity, it is possible to overcome this problem by applying a morphological closing (Fig. 3(f)). False detections due to noise and clutter are removed by merging the binary image with a mask, i.e. all pixels which are zero in the mask are set to zero (Fig. 3(g)). The mask itself is calculated from the segmentation result 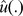 (Fig. 3(b)). First large clutter such as apoptotic cell nuclei, i.e. dead nuclei, are removed from 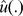, by applying a morphological opening, and then using the inverse of this opened image as a mask on 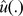 (Fig. 3(c)).

Next, a set of potential nuclei are detected by applying the connected component algorithm on the binary image. Finally, for each potential nucleus, i.e. connected component, a score is calculated. This score corresponds to the mean intensity in the preprocessed image of the pixels corresponding to the connected component in the 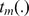 image (Fig. 3(h)). The potential nucleus with the strongest score, 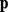, is removed from the potential list and is added to the list of detections. Since nuclei can only touch each other (they can not occupy the same space), all potential nuclei within a distance of the dominant radius of 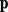 can be removed from the list of potential nuclei. The steps of adding the strongest potential nucleus to the list of detections and removing “too close” potential nuclei are repeated until the list of potential nuclei is empty.

### 0.5 Validation metrics

In order to validate the proposed method we use a number of validation metrics. For the validation of cell nuclei detection we use the following three metrics:

- Root mean squared error (RMSE) is an error measure on the count of cell nuclei: 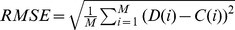, with 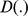 the number of detected nuclei in the 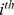 image of the data-set, whereas 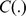 represents the real number of nuclei in the 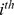 image of the data-set.
- Precision (P) is the ratio of the number of correct detected nuclei (TP), over the total number of detected nuclei, i.e. including false positives.
- Recall (R) is the ratio of the number of TP's in an image, over the total number of nuclei in the ground truth.
- the F-measure takes both false positives and false negatives into account by combining precision and recall: 
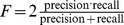
.

All three measures have a value near one for good detection results and a value near zero for bad detection results. A detected nucleus is considered a true positive if there is a ground truth centroid within a range of 3.2 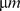. A ground truth nucleus can only be matched with a single detection, if more nuclei detections are within the range of 3.2 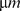 only the closest detection is considered as correctly detected.

For the validations of the segmentation, the Dice coefficient is used. Consider 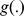, a binary image corresponding to the ground truth, i.e. all foreground pixels are equal to one, whereas all background pixels are equal zero. Then the Dice coefficient between the binarized segmentation result, 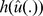, and the ground truth, 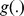, is defined as:
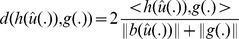
(21)If 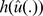 and 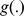 are equal, the Dice coefficient is equal to one. Note that the Dice coefficient is calculated between full images and not for each nucleus independently.

## Results and Discussion

**Figure 4:**
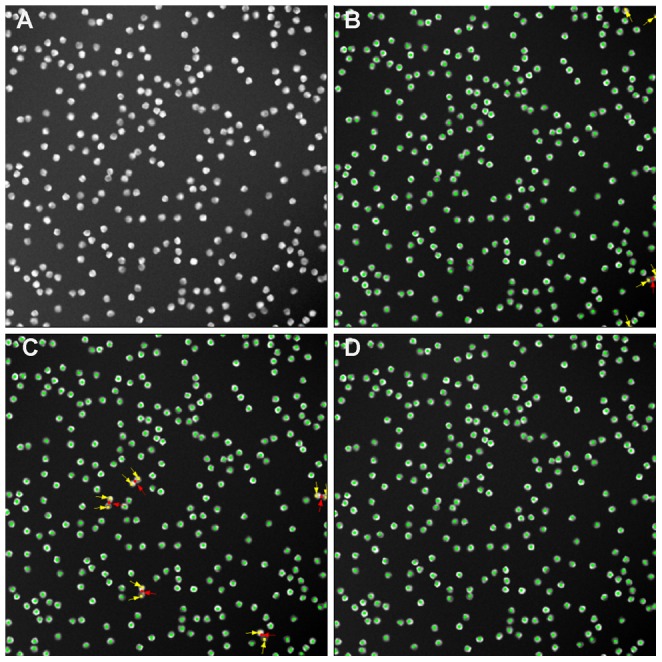
An example of cell nuclei detection using different methods on a benchmark image. (A) the original raw micrograph, (B)–(D) show the respective detection results superimposed on the preprocessed images. (B) edgeProp, (C) cellProfiler, (D) the proposed method. Correctly detected nuclei are shown with a green marker, errors are marked with an arrow: false detections with a red marker and cell nuclei which were not detected by the method are shown using a yellow marker. While edgeProp is able to separate clusters of nuclei, they are limited by the strength of the edges, resulting in several nuclei which are not detected.

As a first validation of the proposed method, a synthetic data set is analyzed [30]. These synthetic images were proposed as a common benchmark for nuclei segmentation and detection. The synthetic images show the same intrinsic properties as real microscopic images of cell nuclei: blurred nuclei, non uniform intensity within in a nucleus, touching nuclei, non uniform background, etc. In Fig. 4(a) an example of such a synthetic raw image is shown. For these experiments, the parameters for eq. are empirically chosen: 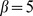, 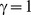, with a disk diameter equal to 20 pixels. The expected intensities needed in eq. are 11 estimated based on the result of a simple Otsu thresholding [31], [32]. Fig. 4(b) shows the detection result using an edge based detection method [10]. The result of cellProfiler, which is a very popular example of an intensity based method [11], is shown in Fig. 4(c), whereas Fig. 4(d) depicts nuclei detection using the proposed method. Correctly detected nuclei are shown with a green marker, errors are marked with an arrow: false detections with a red marker and cell nuclei which were not detected by the method are shown using a yellow marker. While edgeProp is able to separate clusters of nuclei, the method is limited by the strength of the edges, resulting in several nuclei which are not detected. CellProfiler, an intensity based method, is able to detect all nuclei clusters, but is not always able to separate them into individual nuclei. This can be seen by the occurrence of a red marker, a false detection, in the vicinity of multiple yellow markers, i.e. undetected nuclei. Clearly, the proposed method is more robust for detecting clusters of nuclei, while remaining able to detect all nuclei, even those with weak edges.

**Figure 5:**
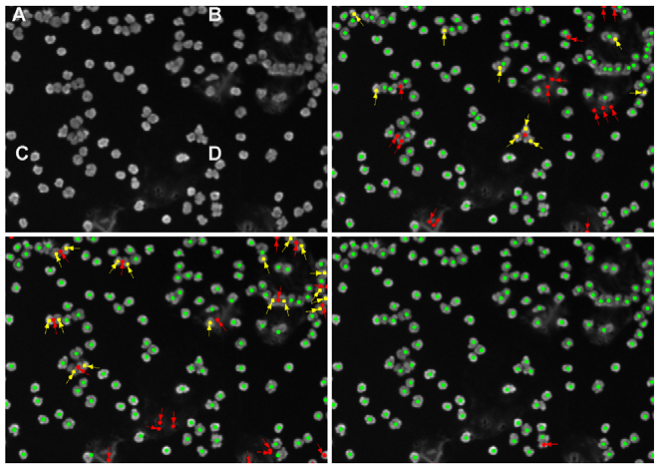
Example of cell nuclei detection using different methods on an image of peripheral blood mononuclear cells. (A) the original raw micrograph, (B)–(D) show the respective detection results superimposed on the preprocessed images. (B) edgeProp, (C) cellProfiler, (D) the proposed method. Correctly detected nuclei are shown with a green marker, errors are marked with an arrow: false detections with a red marker and cell nuclei which were not detected by the method are shown using a yellow marker. While edgeProp is able to separate clusters of nuclei, they are limited by the strength of the edges, resulting in several nuclei which are not detected.

To quantitatively validate the result, a data set of 20 images, each containing 300 nuclei, is analyzed. The results are shown in Table 1. The first row represents the results of an edge based method [10], whereas the next two rows correspond with two intensity based images [11], [12]. The proposed method, presented in the last row, shows the best results for cell detection metrics (first 5 columns) as well as for the Dice coefficient. Note that lacking ground truth for individual nuclei, the Dice coefficient measures the similarity of all segmented nuclei compared with the ground truth for all nuclei instead of for individual nuclei. While state-of-the-art methods perform reasonably for the nuclei count and detection part, the Dice coefficient shows still room for improvement. These good results are also due to the nature of this data-set, which nicely models microscopic image degradations, but where the objects are not hampered by biological clutter, such as dead cells. A second validation is done using real microscopic images of nuclei from peripheral blood mononuclear cells (PBMCs). PBMC's consist of a number of different cell types, each with different nuclei morphologies: 75% lymphocytes (T cells, B cells, NK cells), monocytes, macrophages (rarely), basophils, dendritic cells, neutrophils (horse-shaped nucleus), eosinophils,…) Ground truth for these images is generated by manually annotating the data sets. In Fig. 5 an example of nuclei detection in such a real microscopic image is shown. Fig. 5.a shows the ground truth detection, i.e. the manual annotations. Fig. 5.b and Fig. 5.c correspond to state-of-the-art detection methods [10], [11]. Note that both methods erroneously detect nuclei at places where there is some smeared staining. When a nucleus ruptures it releases its staining, which results in bright smears. CellProfiler does not only suffer from false detections, but also merges touching nuclei. The proposed method is more robust against dye smears, while still being able to detect touching nuclei, as can be seen in Fig. 5.d.

**Table 1:** A comparison of different cell nuclei detection and segmentation methods on the BBBC004v1 data set from the Broad Bioimage Benchmark CollectionA comparison of different cell nuclei detection and segmentation methods on the BBBC004v1 data set from the Broad Bioimage Benchmark Collection.

	RMSE	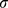	P	R	F	Dice
EdgeProp	1.75	1.78	0.9973	0.9968	0.9971	0.941
CellProfiler	3.79	2.16	0.9914	0.9810	0.9862	0.939
CellC	16.24	3.63	0.9445	0.9945	0.9689	0.929
Proposed	*0.22*	*0.22*	*0.9998*	*1.0000*	*0.9999*	*0.981*

Three different data sets were analyzed, each data set with a different density of cell nuclei (Table 2). The first data set contained approximately 30% of touching nuclei, the second data set with almost 60% of the nuclei touching each other and finally a dense data set with 75% of clustered nuclei. For these experiments, the parameters for eq. are empirically chosen: 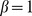, 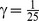, with a diameter in the range of [7.04 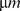
–12.16 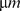]. The expected intensities needed in eq. are 11 estimated based on the result of a simple Otsu thresholding [31], [32]. State-of-the-art methods use the same prior knowledge, i.e. the range of diameters of desirable segments. The actual configuration files for CellC, EdgeProp and CellProfiler can be downloaded at http://telin.ugent.be/jdvylder/nuclei_dictionary/index.htm. The state-of-the-art methods significantly decrease in accuracy of the detections if the micrographs are more densely packed with cell nuclei (Table 3). From the state-of-the-art techniques the edge based approach is generally more robust against touching nuclei, but still performs significantly less than the proposed method. The method presented in this work not only performs best for relatively sparse spaced nuclei, i.e. 30% of touching nuclei, but also for densely clustered nuclei. The fluorescent dye also remains more compact in dense clusters of nuclei, since there is less free space to dissolve the dye.

**Table 2:** ground truth statistics of the different data sets.

	data set 1	data set 2	dataset3
# nuclei	3178	3197	582
# clusters	1025	1861	436

**Table 3:** Comparison of the detection results from different state-of-the-art methods for nuclei of peripheral blood mononuclear cells.

	data set 1			data set 2			data set 3		
method	P	R	F	P	R	F	P	R	F
EdgeProp	0.823	0.895	0.858	0.774	0.847	0.809	0.649	0.801	0.730
CellC	*0.956*	0.824	0.885	0.880	0.694	0.776	0.717	0.488	0.581
CellProfiler	0.855	0.953	0.902	0.794	0.887	0.838	0.500	0.605	0.547
Proposed	0.948	*0.969*	*0.959*	*0.905*	*0.969*	*0.936*	*0.801*	*0.950*	*0.870*

**Figure 6:**
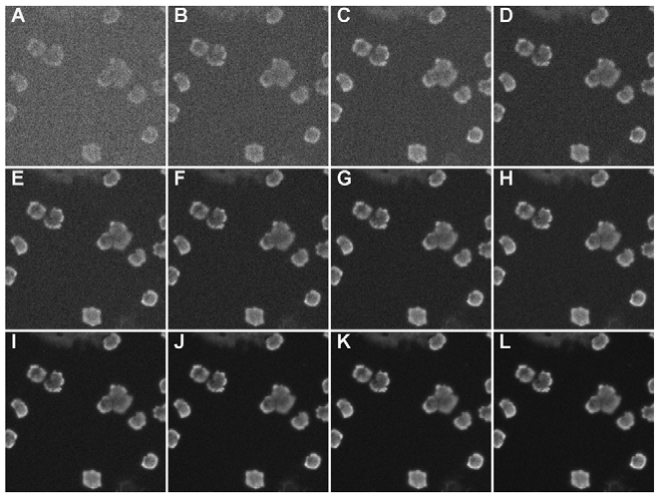
An example of the influence of capturing time on a microscopic image. These images are captured using respectively 5, 10, 20, 30, 40, 50, 75, 100, 200, 300, 400 and 500 ms.

In a third experiment we test the robustness against low exposure times. The influence of the capturing time on the quality of the image is illustrated by an example in Fig. 6. For visual inspection by an expert, an exposure time of 200 ms is advisable. This results in good contrast and low noise images. However for automatic detection lower exposure times also give adequate results, resulting in faster scanning times, lower influence of phototoxicity and less risk on photobleaching [2]. The ideal exposure time depends on the required accuracy and on the application, e.g. in vivo experiments will require lower scanning times due to phototoxicity. State-of-the-art methods clearly lack robustness against low exposure times (Table 4), while the proposed method shows accurate detection results, even for low exposure times. For an exposure time of 30 ms, 15% of the 200 ms used for human operators, the proposed method still results in an F-measure of 0.861. All processing work was performed in Matlab R20007b1 on an Intel i7 Q720 1.6 GHz CPU processor with 4 GB memory. It took an average of 26.944 s for the analysis of a single image from the BBBC004v1 data set from the Broad Bioimage Benchmark Collection, i.e. for the analysis of an image of 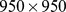 pixels. While this method is computationally more demanding than state-of-the-art methods (cellC, cellprofiler and edgeProp require respectively 4.33 s, 5.80 s and 9.78 s)), the difference is not sufficient to warrant exclusion from practical use. Furthermore the analysis of a data set can be offloaded to a dedicated server. By applying such a pipeline architecture, the bottleneck from the image processing work flow is moved from the acquisition, using expensive microscopes, to the analysis on an inexpensive computer.

**Table 4:** Comparison of the detection results from different state-of-the-art methods for nuclei of peripheral blood mononuclear cells captured using different exposure times.

	EdgeProp			CellC			CellProfiler			Proposed		
exposure time (ms)	P	R	F	P	R	F	P	R	F	P	R	F
5	0.288	0.560	0.381	0.053	0.028	0.037	0.017	0.291	0.033	*0.476*	*0.679*	*0.560*
10	0.384	0.662	0.486	0.030	0.045	0.036	0.030	0.633	0.057	*0.673*	*0.835*	*0.745*
20	0.467	0.769	0.581	0.256	0.148	0.188	0.097	0.744	0.171	*0.790*	*0.899*	*0.841*
30	0.529	0.778	0.630	0407	0.297	0.343	0.196	0.778	0.313	*0.815*	*0.912*	*0.861*
40	0.524	0.778	0.626	0.548	0.387	0.454	0.277	0.829	0.402	*0.799*	*0.906*	*0.849*
50	0.578	0.793	0.669	0.616	0.393	0.480	0.334	0.833	0.477	*0.826*	*0.931*	*0.875*
75	0.576	0.774	0.661	0.810	0.440	0.570	0.416	0.829	0.554	*0.831*	*0.925*	*0.875*
100	0.577	0.759	0.656	*0.861*	0.432	0.576	0.451	0.840	0.587	0.836	*0.932*	*0.882*
200	0.634	0.761	0.692	0.853	0.424	0.567	0.496	0.855	0.628	*0.872*	*0.923*	*0.897*
300	0.629	0.743	0.681	*0.880*	0.455	0.600	0.496	0.829	0.620	0.874	*0.923*	*0.898*
400	0.643	0.741	0.688	0.888	0.464	0.610	0.532	0.870	0.660	*0.892*	*0.931*	*0.911*
500	0.640	0.733	0.684	0.875	0.461	0.603	0.519	0.853	0.645	*0.889*	*0.931*	*0.909*
1000	0.670	0.729	0.700	0.887	0.485	0.627	0.545	0.868	0.670	*0.896*	*0.921*	*0.908*

All data used in these experiments is publicly available at http://telin.ugent.be/jdvylder/nuclei_dictionary/index.htm.

## Conclusion

This paper proposed a novel computer vision technique to detect and segment cell nuclei in fluorescent microscopic images. The method fits within the active contour framework and has a convex energy function. The method uses prior knowledge about the shape of cell nuclei, which is done by representing the segmentation result using a dictionary. The proposed method was tested both on a benchmark data set and on real microscopic data sets of nuclei belonging to peripheral blood mononuclear cells, showing generic value for the detection of nuclei with an approximately circular shape. The method results in accurate nuclei detection and outperforms state-of-the-art methods, both for precision, recall, F-measure and Dice coefficient. The results show that the method is highly robust against dense cell nuclei clusters and can be used for noisy images captured using low exposure times.
